# Low molecular weight heparin versus other anti-thrombotic agents for prevention of venous thromboembolic events after total hip or total knee replacement surgery: a systematic review and meta-analysis

**DOI:** 10.1186/s12891-018-2215-3

**Published:** 2018-09-08

**Authors:** Xin Lu, Jin Lin

**Affiliations:** Department of Orthopaedics, Peking Union Medical College Hospital, Chinese Academy of Medical Sciences and Peking Union Medical College, Beijing, China

**Keywords:** Low molecular weight heparin, Total knee replacement, Total hip replacement, Bleeding, Thrombosis

## Abstract

**Background:**

Venous thromboembolism (VTE) is an important complication following total hip replacement (THR) and total knee replacement (TKR) surgeries. Aim of this study was to comprehensively compare the clinical outcomes of low-molecular-weight heparin (LMWH) with other anticoagulants in patients who underwent TKR or THR surgery.

**Methods:**

Medline, Cochrane, EMBASE, and Google Scholar databases were searched for eligible randomized controlled studies (RCTs) published before June 30, 2017. Meta-analyses of odds ratios were performed along with subgroup and sensitivity analyses.

**Results:**

Twenty-one RCTs were included. In comparison with placebo, LMWH treatment was associated with a lower risk of VTE and deep vein thrombosis (DVT) (*P* values < 0.001) but similar risk of pulmonary embolism (PE) (*P* = 0.227) in THR subjects. Compared to factor Xa inhibitors, LMWH treatment was associated with higher risk of VTE in TKR subjects (*P* < 0.001), and higher DVT risk (*P* < 0.001) but similar risk of PE and major bleeding in both THR and TKR. The risk of either VTE, DVT, PE, or major bleeding was similar between LMWH and direct thrombin inhibitors in both THR and TKR, but major bleeding was lower with LMWH in patients who underwent THR (*P* = 0.048).

**Conclusion:**

In comparison with factor Xa inhibitors, LMWH may have higher risk of VTE and DVT, whereas compared to direct thrombin inhibitors, LMWH may have lower risk of major bleeding after THR or TKR.

**Electronic supplementary material:**

The online version of this article (10.1186/s12891-018-2215-3) contains supplementary material, which is available to authorized users.

## Background

Venous thromboembolism (VTE) is an important complication following total hip replacement (THR) and total knee replacement (TKR) surgeries. The risk of postoperative thromboembolic events was estimated to be approximately 50% for an asymptomatic event and 15% to 30% for a symptomatic event in the absence of prophylactic treatment [1, 2]. These procedures can also result in deep venous thrombosis (DVT), pulmonary embolism (PE), infection, and death [3]. Asian patients aged ≥40 years had a significantly higher relative risk of developing DVT, proximal DVT and PE [4].

Anticoagulants are routinely used and recommended after major orthopedic surgery to prevent VTE Anticoagulants has been found to reduce the risk of thromboembolic events by approximately 50% to 80% when prescribed prophylactically [1]. Both the American College of Chest Physicians (ACCP) and American Association of Orthopedic Surgeons (AAOS) guidelines for VTE prophylaxis recommend antithrombotic prophylaxis following THR or TKR [2, 4]. However, although pharmacologic thromboprophylaxis in patients with THR or TKR may decrease the incidence of VTE and other thrombus related events, it can cause increased risk of major bleeding [2, 5]. A strong relationship between major bleeding and poor outcome irrespective of the study drug used has been demonstrated [6]. Hence, the trade-offs between fewer symptomatic PE and DVT with thromboprophylaxis versus increased major bleeding should be considered [2, 7].

Current guidelines for thromboprophylaxis recommend the use of vitamin K antagonists (e.g. warfarin), low-molecular-weight heparins (LMWH), aspirin, or indirect inhibitor of factor Xa [8]. The efficacy and safety of LMWH is well established [5, 9]. It has a long half-life with good bioavailability [9] and is administered once daily subcutaneous dose without laboratory monitoring or dose adjustment. It is safe and effective for extended out-of-hospital prophylaxis after TKR or THR surgery [10]. Disadvantages associated with LMWH include parenteral administration, expense, potential thrombocytopenia, and poor patient adherence [11, 12]. In a previous meta-analysis, patients who received LMWH (e.g. enoxaparin) prophylaxis had lower incidence of DVT after knee arthroscopic surgery compared to patients who did not receive LMWH prophylaxis [13].

New generation of oral anticoagulants, such as dabigatran etexilate, ximelagatran, rivaroxaban, and apixaban, are now available for prophylaxis against VTE in patients undergoing TKR or THR surgery [14]. Factor Xa inhibitors (i.e., rivaroxaban, darexaban, and apixaban) and direct thrombin inhibitors (i.e., ximelagatran and dabigatran etexilate) have more predictable anticoagulant effects compared to LMWH which also overcome the need to monitor patients receiving short-term thromboprophylaxis [6]. However, disadvantages associated with these drugs include costs and lack of antidotes for timely reversal of bleeding [6].

Currently, there is no comprehensive review to summarize the relative effectiveness of LMWH by comparing it with placebo, factor Xa inhibitors or direct thrombin inhibitors in preventing VTE and incidence of major bleeding when used as thromboprophylaxis agent in TKR or THR surgical interventions. The aim of this meta-analysis was to assess the in-patient clinical outcomes of LMWH compared to factor Xa inhibitors and direct thrombin inhibitors in TKR or THR surgery subjects.

## Methods

### Search strategy

The study was performed in accordance with the PRISMA guidelines. Following databases were searched for studies published before June 30, 2017: Medline, Cochrane, EMBASE, and Google Scholar. The search term (Hip OR Knee), (replacement OR arthroplasty), (low molecular weight heparin OR enoxaparin), (Venous Thromboembolism OR Pulmonary Embolism OR Vein Thrombosis) AND (inhibitor of factor Xa OR direct thrombin inhibitor) and Randomized controlled trial (RCTs) were used.

### Eligibility

Eligible studies had to have investigated patients undergoing hip or knee arthroplasty or replacement, and to have compared patients receiving LMWH (enoxaparin) with placebo, factor Xa inhibitors or direct thrombin inhibitors. Included studies had to have reported outcomes of interests (given below). Retrospective studies, one arm studies, letters, commentaries, editorials, case reports, proceedings, and personal communications were excluded. Also excluded were studies that evaluated anticoagulants other than direct thrombin and factor Xa inhibitors (e.g. aspirin or warfarin).

### Quality assessment

The quality of the included studies was assessed using Quality in Prognostic Studies (QUIPS), which consists of six domains (study participation, study attrition, prognostic factor measurement, outcome measurement, confounding measurement and account, analysis) [15, 16].

### Data and statistical analysis

The following information/data was extracted from studies that met the inclusion criteria: the name of the first author, year of publication, study design, number of participants in each group, participants’ age and gender, and major outcomes. The outcomes of interest were the risk or odds of thrombotic events (VTE, DVT, PE, major bleeding). Basic characteristics of the included studies were summarized as mean ± standard deviations (SD), mean (range: min., max.), or median (min., max.) for age, and n (%) for gender and patient number. The outcomes were summarized as n/N (patients with events out of total number of patients) for given intervention as LMWH vs controls (placebo, or factor Xa inhibitor, or a direct thrombin inhibitor). When assessment of an outcome included ≥3 studies, an effect size odd ratio (OR) with corresponding 95% confidence intervals (95% CI) was calculated for each individual study and then overall effect size was generated . Meta-analyses was not performed when ≤2 studies reported an outcome of interest. Odds ratios > 1 implied patients with LMWH treatment had a higher rate of a given outcome than those treated with control; OR < 1 indicated patients with LMWH treatment had a lower rate of a specific outcome than patients receiving control therapy; OR = 1 suggested the rate of an outcome was similar between LMWH and control treatments.

A χ^2^ test for homogeneity was conducted, and an inconsistency index (I^2^) and Q statistics were determined [17]. If the I^2^ statistic was > 50%, a random-effects model (Der Simonian–Laird method) was used [18]. Otherwise, a fixed-effects model (Mantel-Haenszel method) was employed. Combined effects were calculated, and a two-sided *P* value of < 0.05 was considered significant. Sensitivity analyses were performed using a leave-one-out approach. Publication bias was assessed as guided by the Cochrane Handbook for Systematic and summarized using Review Manager Software (Version 5.3). However, the funnel plot and Egger’s test were not performed because the limitation of the study numbers (≤10 per outcome) [19]. All data were organized in Microsoft Office Excel 2007 spread sheets and all meta-analyses were performed using Comprehensive Meta-Analysis statistical software, version 2.0 (Biostat, Englewood, NJ, USA). Safety analyses were performed with Stata software (version 12, Stata Corporation, Texas, USA).

## Results

### Search results

**Fig. 1:**
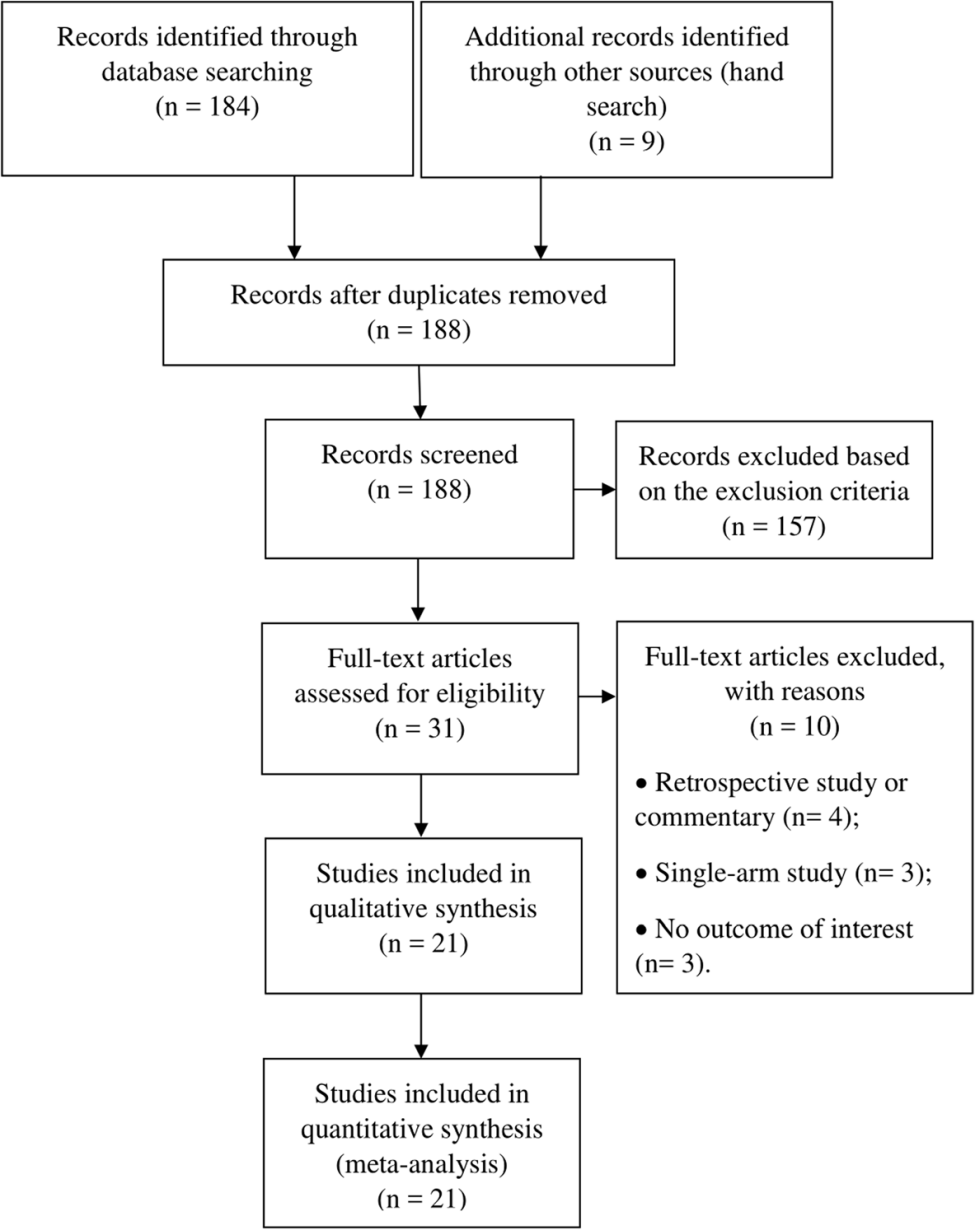
Flow Diagram for study search

A total of 184 articles were identified through database searches and nine through corroborative searches (Fig. 1). After removing duplicates and an initial screen of abstracts and titles to remove studies that did not meet the inclusion criteria, 31 studies underwent full text review. Subsequently, 10 articles were excluded due to the ineligible design (retrospective study or commentary) (*n* = 4), being a single-arm study (*n* = 3), and not reporting outcome of interest (*n* = 3). Consequently, 21 studies were included in the systematic review and meta-analysis [10, 20–39].

### Characteristics of included studies

The studies were divided into three subgroups based on the non-LMWH treatment: Group I: LMWH vs. placebo (3 studies); Group II: LMWH vs. direct thrombin inhibitors (8 studies; 4 studies with ximelagatran and 4 with dabigatran etexilate); Group III: LMWH vs. factor Xa inhibitor (10 studies; 6 with studied rivaroxaban, 3 with apixaban, and 1 with darexaban). The study of Kim et al. (2016) had two control groups: rivaroxaban group and placebo group [20]. Therefore, the data from Kim et al. [20] for LMWH vs. placebo were included in meta-analysis of Group I. Group I included 962 participants (708 for LMWH and 254 for placebo), Group II included 18,116 participants (7530 for LMWH and 10,586 for control), and Group III included 26,639 participants (12,713 for LMWH and 13,926 for control). The mean ages for most studies were in general ≥60 years, except for Kim et al. [20] which participants had mean age of about 43 to 44 years. Male patients ranged from 6% [29] to 62% [20]. Mean body mass index (BMI) ranged from 23.5 kg/m^2^ [29] to 32.6 kg/m^2^ [10]. Details are provided in Table 1.

**Table 1:** Patients’ characteristics among the studies

1st author (year)	Treatments	Details of treatment (dose and routes of Administration)	number of patients	Procedure (n)	Age mean ± SD years	Sex, Males (%)	BMI, kg/m^2^	Follow-up time (days)
I. *LMWH* vs. *placebo (saline)*								
Fuji (2008) [10]	Enoxaparin	20 mg sc qd for 14d	81	THR	63.3 ± 10.4	10 (12.3)	23.5 ± 3.4	90
		40 mg sc qd for 14d	80		60.6 ± 9.9	6 (7.5)	23.5 ± 3.7	
		20 mg sc bid for 14d	90		63.0 ± 9.3	15 (16.7)	23.7 ± 3.6	
	Placebo	Saline bid for 14d	86		62.0 ± 10.3	11 (12.8)	24.0 ± 3.4	
	Enoxaparin	20 mg sc qd for 14d	78	TKR	68.8 ± 9.0	15 (19.2)	25.7 ± 4.5	90
		40 mg sc qd for 14d	74		70.0 ± 9.4	11 (14.9)	25.3 ± 4.0	
		20 mg sc bid for 14d	84		68.3 ± 8.7	5 (6)	24.0 ± 4.0	
	Placebo	Saline bid for 14d	79		68.7 ± 9.5	15 (19)	25.4 ± 3.7	
Bergqvist (1996) [20]	Enoxaparin	40 mg (0.4 ml) sc qd	131	THR	median = 70 (range: 44–87)	56 (42.7)	median = 25.8 (range:18.6–37.9)	n/a
	Placebo	0.4 ml saline qd	131		median = 70 (range: 44–87)	57 (43.5)	median = 26.8 (range: 19.2–49.1)	
Planes (1996) [21]	Enoxaparin	40 mg sc qd for 2d	90	THR	70 ± 9·1	47 (52.2)	25·55 ± 3·19	21
	Placebo	Saline qd	89		68 ± 8·2	55 (61.8)	25·85 ± 3·49	
II. *LMWH* vs. *inhibitor of factor Xa*								
Kim (2016) [1]	Enoxaparin	40 mg sc qd for 14d	184	THR	43.9 ± 9.4	114 (62.0)	25.4 ± 3.8	N/A
	Rivaroxaban	10 mg qd for 14d	184		44.4 ± 8.6	99 (53.8)	24.8 ± 3.1	
	Placebo	1 ml saline or placebo tablet qd	185		43.4 ± 9.9	109 (58.9)	24.3 ± 3.4	
Zou (2014) [3]	Enoxaparin	4000 anti-Xa IU (0.4 ml) qd for 14d	112	TKR	mean = 65.7 (range:54–80)	20 (17.85)	mean = 27.0 (range:20.3–37.0)	28
	Rivaroxaban	10 mg qd for 14d	102		mean = 63.5 (range:50–82)	32 (31.37)	mean = 27.5 (range:18.0–39.5)	
	Aspirin	100 mg qd for 14d	110		mean = 62.7 (range:47–79)	28 (25.45)	mean = 27.8 (range:17.8–40.0)	
Kakkar (2008) [RECORD 2] [11]	enoxaparin	40 mg sc qd for 10–14 d (with placebo tablet for 31–39d)	1229	THR	61.6 ± 13.7	578 (47)	27.1 ± 5.2	n/a
	Rivaroxaban	10 mg qd 31–39 d (with placebo injection for 10–14 d)	1228		61.4 ± 13.2	561 (45.7)	26.8 ± 4.8	
Eriksson (2008) [RECORD 1] [12]	Enoxaparin	40 mg sc qd for 35d (range, 31-39d) (with placebo tablet)	2224	THR	median = 63.3 (range:18–93)	982 (44.2)	median = 27.9 (range:15.2–50.2)	30–42
	Rivaroxaban	10 mg qd for 35d (range, 31-39d) (with placebo injection)	2209		median = 63.1 (range:18–91)	989 (44.8)	median = 27.8 (range:16.2–53.4)	
Lassen (2008) [RECORD 3] [13]	Enoxaparin	40 mg sc qd for 10-14d	1239	TKR	median = 67.6 (range:30–90)	419 (33.7)	median = 29.8 (range:16.0–54.3)	30–35
	Rivaroxaban	10 mg qd for 10-14d	1220		median = 67.6 (range:28–91)	363 (29.8)	median = 29.5 (range:16.3–51.1)	
Turpie (2009) [RECORD 4] [8]	Enoxaparin	30 mg sc bid for 10-14d	1508	TKR	64.7 ± 9.7	541(35.9)	30.7 ± 6.0	30–35
	Rivaroxaban	10 mg qd for 10-14d	1526		64.4 ± 9.7	519 (34.0)	30.9 ± 6.2	
Lassen (2009) [ADVANCE-1] [9]	Enoxaparin	30 mg sc bid for 10-14d (with placebo tablet)	1596	TKR	mean = 65.7 (range: 33–89)	610 (38.2)	mean = 31.1 (range:17.7–57.6)	60
	Apixaban	2.5 mg bid for 10-14d (with placebo tablet)	1599		mean = 65.9 (range:26–93)	602 (37.6)	mean = 31.2 (range:18.1–57.7)	
Lassen (2010a) [ADVANCE-2] [5]	Enoxaparin	40 mg sc qd for 10-14d (with placebo tablet)	1529	TKR	median = 67 (IQR:60–73)	402 (26)	median = 29.3 (IQR:26.1–32.7)	60
	Apixaban	2.5 mg bid for 10-14d (with placebo tablet)	1528		median = 67 (IQR:59–73)	439 (29)	median = 29.1 (IQR:25.8–32.4)	
Lassen (2010b) [ADVANCE-3] [6]	Enoxaparin	40 mg sc qd for 32-38d (with placebo tablet)	2699	THR	mean = 60.6 (range: 19–93)	1248 (46.2)	mean = 28.1 (range:12.5–48.7)	65 ± 5
	Apixaban	2.5 mg bid for 32-38d (with placebo tablet)	2708		mean = 60.9 (range: 19–92)	1278 (47.2)	mean = 28.2 (range:15.4–58.5)	
Eriksson (2014) [2]	enoxaparin	40 mg sc qd, for 35 d	314/393(ITT)		61.1 ± 11.79	166 (42.2)	28.4 ± 4.66	12
	Darexaban	15 mg bid for 35 d	374/269 (ITT)	THR	60.4 ± 10.95	188 (50.3)	28.4 ± 4.97	
		30 mg qd for 35 d	383/293 (ITT)		59.5 ± 11.73	191 (49.9)	28.8 ± 5.14	
		30 mg bid for 35 d	296/387 (ITT)		59.8 ± 12.13	204 (52.7)	28.5 ± 5.22	
		60 mg qd for 35 d	274/385 (ITT)		59.5 ± 11.82	182 (47.3)	28.9 ± 5.00	
III. *LMWH* vs. *direct thrombin inhibitor*								
Heit (2001) [19]	LMWH	Enoxaparin: 30 mg sc bid for 6-12d	125	TKR	68 ± 10	48 (38)	31.8 ± 7.0	n/a
	Ximelagatran	Ximelagatran	475					
		8 mg for 6-12d	85		65 ± 10	34 (40)	32.6 ± 5.7	
		12 mg for 6-12d	134		67 ± 11	56 (42)	30.9 ± 6.0	
		18 mg for 6-12d	126		68 ± 10	45 (36)	30.9 ± 6.0	
		24 mg for 6-12d	130		67 ± 11	46 (35)	31.4 ± 5.7	
Eriksson (2003); METHRO III study [18]	Enoxaparin	40 mg sc qd started 12 h before surgery for 8-10d	1389	THR (957) TKR (432)	mean = 65.8 (range: 26–93)	549 (40)		n/a
	Ximelagatran	24 mg bid for 8-10d beginning the next day of surgery for 8-10d	1399	THR (966) TKR (433)	mean = 66.4 (range:25–93)	515 (37)		
Colwell (2003) [17]	Enoxaparin	30 mg sc bid for 7-12d	910	THR	64.0 ± 13.1 (*n* = 775)	377 (48.6)	28.3 ± 5.3	n/a
	Ximelagatran	24 mg bid for 7-12d	906		64.5 ± 12.8 (*n* = 782)	372 (47.6)	28.4 ± 5.3	
Eriksson (2003); EXPRESS study [16]	Enoxaparin	40 mg sc qd started 12 h before surgery for 8-10d	1387 (ITT)	THR (942) TKR (445)	median = 67 (range:20–89)	542 (39.1)		n/a
	Ximelagatran	24 mg bid for 8-10d beginning the next day of surgery for 8-10d	1377 (ITT)	THR (914) TKR (463)	median = 67 (range:24–88)	509 (37.0)		
RE-MOBILIZE (2009) [7]	Enoxaparin	30 mg sc bid for 12-15d	868	TKR	66.3 ± 9.6	4364 (1.9)		n/a
	Dabigatran Etexilate	220 mg qd for 12-15d	857		66.2 ± 9.5	371 (43.3)		
		150 mg qd for 12-15d	871		65.9 ± 9.5	364 (41.8)		
Eriksson (2007) a [RE-MODEL] [14]	Enoxaparin	40 mg sc qd for 6-10d	694	TKR	68 ± 9	216 (31)		6–10
	Dabigatran etexilate	220 mg qd for 6-10d	679		67 ± 9	238 (35)		
		150 mg qd for 6-10d	703		68 ± 9	252 (36)		
Eriksson (2007) b [RE-NOVATE; Patients from Europe, Australia, and South Africa] [15]	Enoxaparin	40 mg sc qd for 28-35d	1154	THR	64 ± 11	503 (44)		28–35
	Dabigatran etexilate	220 mg qd for 28-35d	1146		65 ± 10	510 (44)		
		150 mg qd for 28-35d	1163		63 ± 11	496 (43)		
Eriksson (2011) [RE-NOVATE II; Patients from North America] [4]	Enoxaparin	40 mg sc qd for 28-35d (with placebo tablet for 28-35d)	1003	THR	62 ± 11	501 (50)	27.8 ± 4.8	28–35
	Dabigatran etexilate	220 mg qd for 28-35d (with placebo injection for 28-35d)	1010		62 ± 12	541 (53.6)	27.8 ± 4.8	

Abbreviations: bid, twice daily; qd, once daily; *INR* international normalized ratio; *IQR* inter-quartile range; *ITT* intent-to-treat, *IU* international unit, *sc* subcutaneously, *SD* standard deviation, *THA* total hip arthroplasty, *THR* total hip replacement, *TKA* total knee arthroplasty, *TKR* total knee replacement

The drug dose and routes of administration were diverse among the studies. Enoxaparin was the only LMWH used in the included studies at a dose of 40 mg once daily as subcutaneous injection in 15 studies. However, for five studies, the postoperative regimen of 30 mg of enoxaparin administered subcutaneously every 12 h (30 mg bid) was used as this regimen was approved by the Food and Drug Administration (FDA) [21, 26–28, 36]. Zou et al. [22] used enoxaparin sodium as 4000 anti-Xa activity IU (0.4 ml) once daily dose, and Fuji et al. [29] assessed the effectiveness of three different doses of enoxaparin given subcutaneously; 20 mg qd, 40 mg qd, and 20 mg bid. The length of follow-up period ranged from 12 days (Eriksson et al. [21]) to 90 days (Fuji et al. [29]).

The efficacy and safety outcomes including rate of total VTE, DVT, PE, major bleeding, and clinical relevant non-major bleeding or minor bleeding are summarized in Table 2. In placebo- controlled studies, enoxaparin (LMWH) was associated with lower incidence of major VTE and DVT for both THR and TKR than placebo. In studies in which the effectiveness of enoxaparin was compared with factor Xa inhibitors (i.e., rivaroxaban, apixaban, or darexaban), the incidence of major VTE and DVT in the enoxaparin groups, in general, was lower than in the factor Xa inhibitor groups for both THR and TKR. In studies which compared enoxaparin with direct thrombin inhibitors, enoxaparin appeared to have a higher percentage of patients with major VTE and DVT than ximelagatran but similar or a lower percentage of patients with these events compared with dabigatran etexilate. The percentage of patients with major or minor bleeding for any given treatment appeared to vary across all studies. Results of the meta-analyses are summarized in Additional file 1: Table S1.

**Table 2:** Summary of the efficacy and safety outcomes among the studies

1st author (year)	Treatments	number of patients	Procedure	Efficacy^b^				Safety^b^	
				Death during follow-up	Major VTE (DVT + PE)^a^	DVT	PE	Major bleeding	Minor or non-major bleeding
I. *LMWH* vs. *placebo (saline)*									
Fuji (2008) [29]	Enoxaparin	251	THR		66/251	66/251	0/251	6/306	12/306
	20 mg qd	81			21/81	21/81	–	1/100	1/100
	40 mg qd	80			27/80	27/80	–	2/102	7/102
	20 mg bid	90			18/90	18/90	–	3/104	4/104
	Placebo	86			36/86	36/86	0/86	0/101	2/101
	Enoxaparin	236	TKR		86/236	84/236	2/236	4/275	21/275
	20 mg qd	78			35/78	34/78	1/78	0/89	5/89
	40 mg qd	74			26/74	25/74	1/74	1/91	6/91
	20 mg bid	84			25/84	25/84	0/84	3/95	10/95
	Placebo	79			48/79	48/79	0/79	4/89	4/89
Bergqvist (1996) [38]	Enoxaparin, 40 mg	131	THR	0	21/117	21/117	0/117		
	Placebo	131	THR	0	45/116	45/116	0/116		
Planes (1996) [39]	Enoxaparin, 40 mg	90	THR	0		6/85			
	Placebo	89	THR	0		17/88			
Kim (2016) [20]	Enoxaparin	184	THR	0	11/184	11/184	0/184		
	Rivaroxaban	184		0	11/184	10/184	1/184		
	Placebo	185		0	13/185	12/185	1/185		
Zou (2014) [22]	LMWH	112	TKA			14/112			
	Rivaroxaban	102				3/102			
	Aspirin	110				18/110			
Kakkar (2008) [RECORD 2] [30]	enoxaparin	1229		6/869	75/869	71/869	4/869	1/1229	
	Rivaroxaban	1228	THR	2/864	15/864	14/864	1/864	1/1228	
Eriksson (2008) [RECORD 1] [31]	Enoxaparin	2224	THR	0/1558	33/1678	53/1558	1/1558	2/2224	129/2224
	Rivaroxaban	2209		1/1598	4/1686	12/1598	4/1598	6/2209	128/2209
Lassen (2008) [RECORD 3] [32]	Enoxaparin	1239	TKR	4/1217	24/925	160/878	4/878	6/1239	54/1239
	Rivaroxaban	1220		0/1201	9/908	79/824	0/824	7/1220	53/1220
Turpie (2009) [RECORD 4] [27]	Enoxaparin	1564	TKR	3/1508	22/1112	86/959	8/1508	4/1508	138/1508
	Rivaroxaban	1584		4/1526	13/1122	61/965	4/1526	10/1526	155/1526
Lassen (2009) [ADVANCE-1] [28]	Enoxaparin	1596	THR	3/1554	20/1216	92/1122	7/1596	22/1588	47/1588
	Apixaban	1599		0/1562	26/1269	89/1142	16/1599	11/1596	35/1596
Lassen (2010a) [ADVANCE-2] [24]	Enoxaparin	1529	TKR	1/1469	26/1199	248/997	0/1529	14/1508	58/1508
	Apixaban	1528		1/1458	13/1195	142/971	4/1528	9/1501	44/1501
Lassen (2010b) [ADVANCE-3] [25]	Enoxaparin	2699	TKR	1/2577	25/2195	68/1911	5/2699	18/2659	120/2659
	Apixaban	2708		2/2598	10/2199	22/1944	3/2708	22/2673	109/2673
Eriksson (2014) [21]	enoxaparin	393	THA	0/393	48/314 (ITT)	6/314	1/314	8/393 (2.0)	20/393 (5.0)
	Darexaban	1529		1/1529	150/1132 (ITT)	9/1132	4/1132	25/1529 (1.6)	61/1529 (4.0)
	15 mg bid	374			42/269 (ITT)	3/269	2/269	5/374	
	30 mg qd	383			39/293 (ITT)	1/293	1/293	4/383	
	30 mg bid	387			33/296 (ITT)	2/296	1/296	9/387	
	60 mg qd	385			36/274 (ITT)	3/274	0/274	7/385	
II. *LMWH* vs. *direct thrombin inhibitor*									
Heit (2001) [10]	Enoxaparin	125	TKR	0/125	23/97	23/97	0/97	1/125	
	Ximelagatran	475	TKR	1/475	77/447	74/447	3/447		
	8 mg	85			17/63	17/63	0/63	0/85	
	12 mg	134			20/202	20/202	0/202	0/134	
	18 mg	126			25/87	23/87	2/87	2/126	
	24 mg	130			15/95	14/95	1/95	0/130	
Eriksson (2003b) [37]	Enoxaparin	957	THR	0/957^c^	45/823			10/942	61/942
	Ximelagatran, 24 mg	966		4/966^c^	14/773			37/915	87/915
	Enoxaparin	432	TKR		29/355			6/445	36/445
	Ximelagatran, 24 mg	433			12/365			9/463	39/463
Colwell (2003) [36]	Enoxaparin	910			36/775	36/775	0/775	8/910	
	Ximelagatran, 24 mg	906	THR		62/782	62/782	0/782	7/906	
Eriksson (2003a) [35]	Enoxaparin	942 (ITT)	THR	0/942^c^	146/801	142/801	4/801	10/942	
	Ximelagatran, 24 mg	914 (ITT)		5/914^c^	99/765	97/765	2/765	37/915	
	Enoxaparin	445 (ITT)	TKR		169/383	167/383	2/383	6/445	
	Ximelagatran, 24 mg	463 (ITT)			132/376	131/376	1/376	9/463	
Ginsberg (2009) [RE-MOBILIZE] [26]	Enoxaparin	868	TKR	0	163/868	158/868	5/868	12 (1.4)	
	Dabigatran Etexilate	1728		2	405/1728	399/1728	6/1728		
	220 mg	857		1	187/857		6/857	5 (0.6)	
	150 mg	871		1	218/871		0/871	5 (0.6)	
Eriksson (2007a) [RE-MODEL] [33]	Enoxaparin	694	TKR	1/685 (0.1)			1/685	9/694 (1.3)	69 (9.9)
	Dabigatran Etexilate	1382		2/763			1/1382		
	220 mg	679		1/675 (0.1)			0/675	10/679 (1.5)	60 (8.8)
	150 mg	703		1/696 (0.1)			1/696	9/703 (1.3)	59 (8.4)
Eriksson (2007b) [RE-NOVATE; Europe, Australia, and South Africa] [34]	Enoxaparin	1154	THR	0/1142	36/917	Asymptomatic: 56/894 Symptomatic: 1/1142	3/1142	18	74
	Dabigatran etexilate	2309		6/2293	66/1797	Asymptomatic: 56/894 Symptomatic: 1/1142	6/2293	38	142
	220 mg	1146	THR	3/1137	28/909	Asymptomatic: 40/874 Symptomatic: 6/1137	5/1137	23	70
	150 mg	1163		3/1156	38/888	Asymptomatic: 63/871 Symptomatic: 9/1156	1/1156	15	72
11Eriksson (2011), 220 mg [RE-NOVATE II; North America] [23]	Enoxaparin	1.003	THR	1/951	4/951	67/783	2/992	9/1003	54/1003
	Dabigatran etexilate	1010		0/942	2/942	60/791	1/1001	14/1010	61/1010

Abbreviations: *DVT* deep vein thrombosis, *PE* pulmonary embolism, *THA* total hip arthroplasty, *THR* total hip replacement, *TKA* total knee arthroplasty, *TKR* total knee replacement, *VET* venous thromboembolism;

^a^Major venous thromboembolism was the composite of proximal deep-vein thrombosis and nonfatal pulmonary embolism

^b^Efficacy and safety were summarized as n/N, that n means number of cases and N means number of given treatments

^c^The number of patients dies was the total death in both THR and TKR groups

### Meta-analysis

#### Venous thromboembolism

**Fig. 2:**
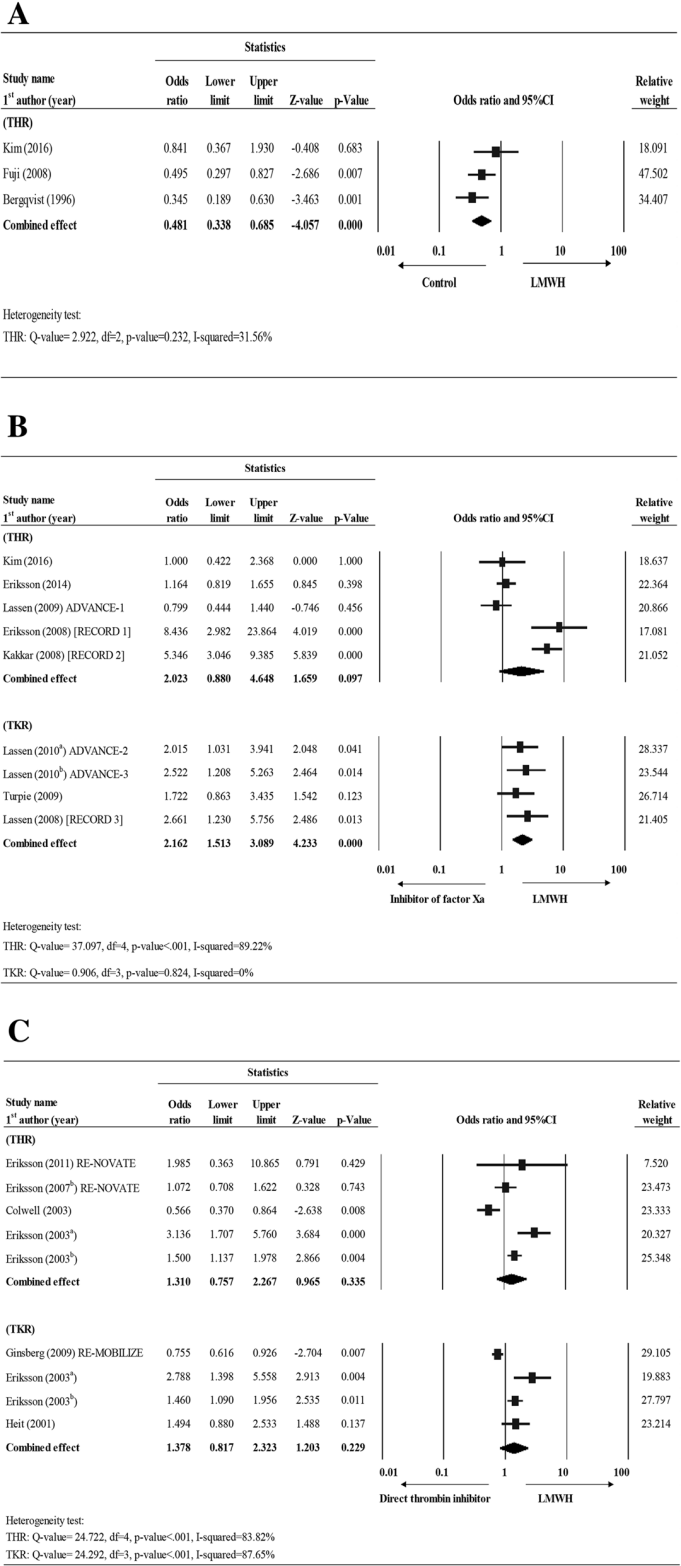
Forest plot for comparing the total VTE rate between (**a**) LMWH vs. control (placebo), (**b**) LMWH vs. inhibitor of factor Xa, and (**c**) LMWH vs. direct thrombin inhibitor for THR and TKR patients. Abbreviations: CI, confidence interval; Lower limit, lower bound of the 95% CI; Upper limit, upper bound of the 95% CI

In Group I (LMWH vs. placebo), three studies reported complete total VTE data for THR [29, 38, 39]. Fixed effect model was used due to low heterogeneity in the data (THR: Q value = 2.922, df = 2, *P* = 0.232, I-squared = 31.56%). The overall effect size showed that the LMWH treatment had significantly lower odds of VTE than the placebo group (OR = 0.481, 95% CI = 0.338–0.685, *P* < 0.001) (Fig. 2a; Additional file 1: Table S1).

In Group II (LMWH vs. inhibitor of factor Xa), nine studies, 5 for THR [20, 21, 28, 30, 31] and 4 for TKR [24, 25, 27, 32], reported complete total VTE data. Random effects model was considered for THR due to the presence of high heterogeneity for the THR data but low heterogeneity for the TKR data, fixed effect model was used (THR: Q value = 37.097, df = 4, *P* < 0.001, I-squared = 89.22%; TKR: Q value = 0.906, df = 3, *P* = 0.824, I-squared = 0%). The overall effect size showed that LMWH group had higher chance of VTE rate than factor Xa inhibitor group for TKR (OR = 2.162, 95% CI = 1.513–3.089, *P* < 0.001) but a similar VTE risk for THR (OR = 2.023, 95% CI = 0.880–4.648, *P* = 0.097) (Fig. 2b; Additional file 1: Table S1).

For Group III (LMWH vs. direct thrombin inhibitor), five studies had complete total VTE data for THR [23, 34–37] and four for TKR [10, 26, 35, 37]. Because data for both THR and TKR findings was heterogenous across the studies (THR: Q-value = 24.722, df = 4, *P* < 0.001, I-squared = 83.82%; TKR: Q-value = 24.292, df = 3, *P* < 0.001, I-squared = 87.65%), hence random effect models were applied. The overall effect size showed that there was no significant difference between LMWH and direct thrombin inhibitors in the incidence of VTE for either THR (OR = 1.31, 95% CI = 0.757–2.267, *P* = 0.335) or TKR (OR = 1.378, 95% CI = 0.817–2.323, *P* = 0.229) (Fig. 2c; Additional file 1: Table S1).

#### Deep vein thrombosis

**Fig. 3:**
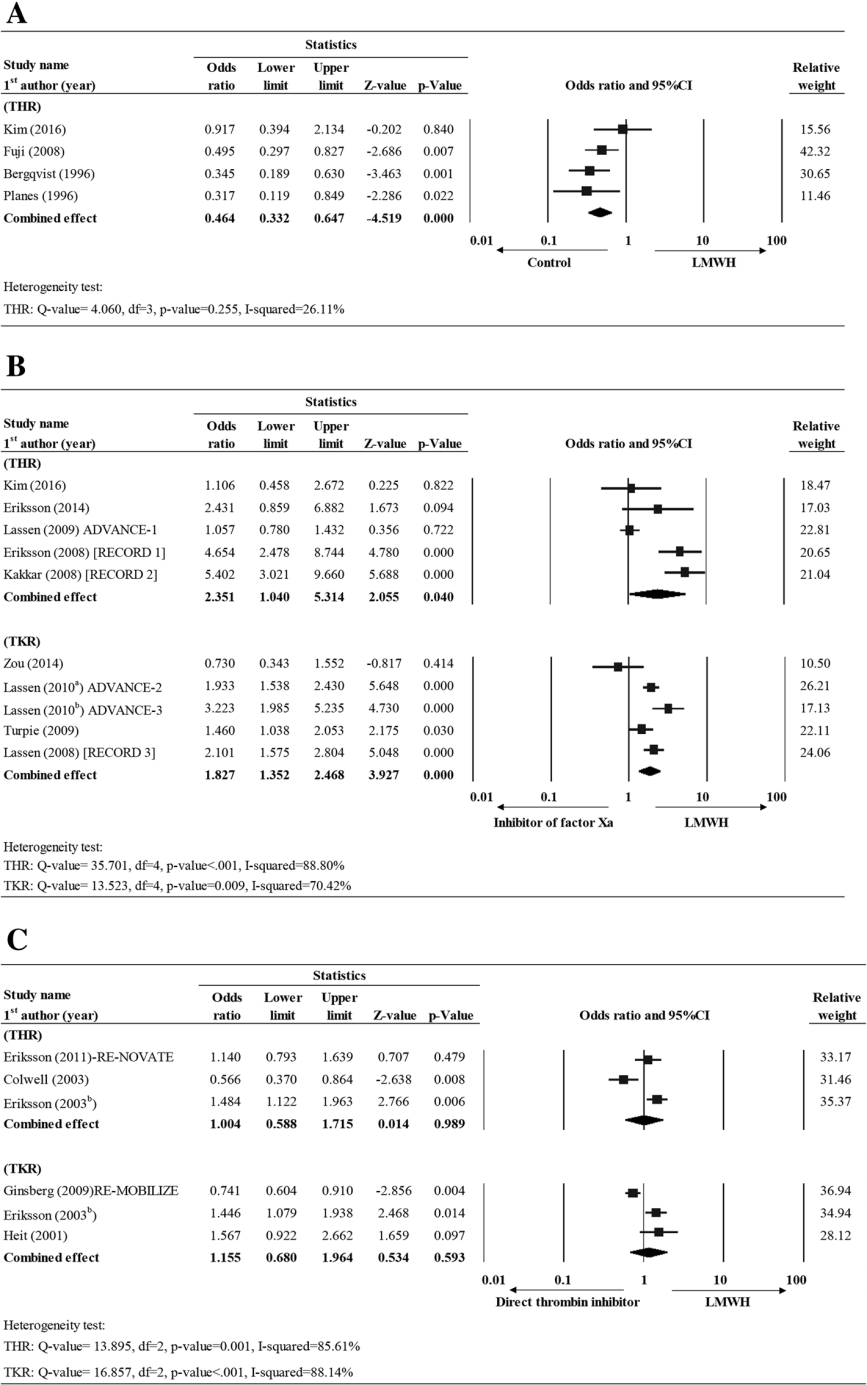
Forest plot for comparing the total DVT rate between (**a**) LMWH vs. control (placebo), (**b**) LMWH vs. inhibitor of factor Xa, and (**c**) LMWH vs. direct thrombin inhibitor for THR and TKR patients. Abbreviations: CI, confidence interval; Lower limit, lower bound of the 95% CI; Upper limit, upper bound of the 95% CI

In Group I (LMWH vs. placebo), four studies reported complete total DVT data for THR subjects [20, 29, 38, 39]. A fixed effect model was used as little heterogeneity in the data was observed (THR: Q value = 4.060, df = 3, *P* = 0.255, I-squared = 26.11%). The overall effect size showed that the LMWH treatment had significantly lower incidence of DVT than placebo group. (OR = 0.464, 95% CI = 0.332–0.647, *P* < 0.001) (Fig. 3a).

In Group II (LMWH vs. inhibitor of factor Xa), ten studies, 5 for THR [20, 21, 28, 30, 31] and 5 for TKR) [22, 24, 25, 27, 32], had complete data for the rate of DVT. A random effects model was used for both THR and TKR due to the presence of high heterogeneity in the data (THR: Q value = 35.701, df = 4, *P* < 0.001, I-squared = 88.80%; TKR: Q value = 13.523, df = 4, *P* = 0.009, I-squared = 70.42%). The overall effect size showed that LMWH group was associated with a higher risk of DVT than factor Xa inhibitors for THR subjects (OR = 2.351, 95% CI = 1.040–5.314, *P* = 0.040) or TKR (OR = 1.827, 95% CI = 1.352–2.468, *P* < .001) (Fig. 3b; Additional file 1: Table S1).

For Group III (LMWH vs. direct thrombin inhibitor), three studies reported complete DVT data for THR [23, 36, 37] and three studies reported full data for TKR [10, 26, 37]. A random effect model was used for both THR and TKR analyses as high heterogeneity was observed in the data across studies (THR: Q-value = 13.895, df = 2, *p*-value = 0.001, I-squared = 85.61%; TKR: Q-value = 16.857, df = 2, *P* < 0.001, I-squared = 88.14%). The overall effect size showed that there was no significant difference between LMWH and direct thrombin inhibitor group in the odds of having a DVT for patients undergoing either THR or TKR (THR: OR = 1.004, 95% CI = 0.588–1.715, *p* = 0.989; TKR: OR = 1.155, 95% CI = 0.680–1.964, *P* = 0.593) (Fig. 3c; Additional file 1: Table S1).

#### Pulmonary embolism

**Fig. 4:**
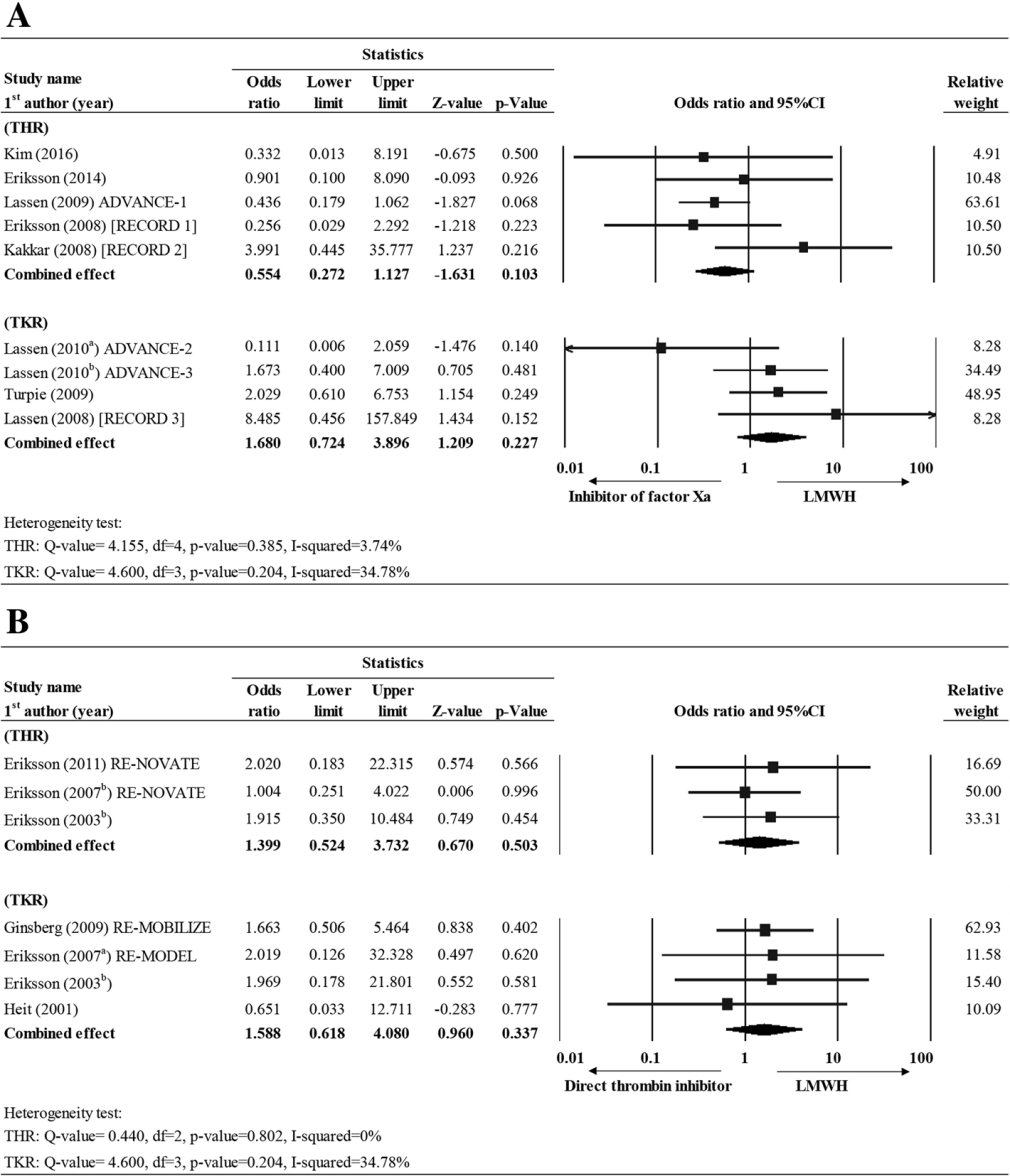
Forest plot for comparing the PE rate between (**a**) LMWH vs. control (placebo), (**b**) LMWH vs. inhibitor of factor Xa, and (**c**) LMWH vs. direct thrombin inhibitor for THR and TKR patients. Abbreviations: CI, confidence interval; Lower limit, lower bound of the 95% CI; Upper limit, upper bound of the 95% CI

Nine studies in Group II (LMWH vs. inhibitor of factor Xa), five for THR [20, 21, 28, 30, 31] and four for TKR [24, 25, 27, 32] reported complete results for PE . Fixed effect model was used as low heterogeneity was observed among the studies for both THR (Q value = 4.155, df = 4, *P* = 0.385, I-squared = 3.74%) and TKR (Q value = 4.600, df = 3, *P* = 0.204, I-squared = 34.78%). The overall effect size showed that LMWH and factor Xa inhibitor group were associated with similar likelihood of developing PE for both THR (OR = 0.554, 95% CI = 0.272–1.127, *P* = 0.10) and TKR. (OR = 1.680, 95% CI = 0.724–3.896, *P* = 0.227) (Fig. 4a, Additional file 1: Table S1).

Three studies in Group III (LMWH vs. direct thrombin inhibitor) reported PE data for THR [23, 33, 34] and four had PE data for TKR [10, 26, 33, 34]. Low heterogeneity was observed for both THR and TKR (THR: Q-value = 0.440, df = 2, *P* value = 0.802, I-squared = 0%; TKR: Q-value = 4.600, df = 3, *P* value = 0.204, I-squared = 34.78%); hence fixed effect model was used for both. The overall effect size showed that LMWH and direct thrombin inhibitor therapies had similar incidence of PE for either THR (OR = 1.399, 95% CI = 0.524–3.732, *P* = 0.503) or TKR (OR = 1.588, 95% CI = 0.618–4.080, *P* = 0.337) (Fig. 4b; Additional file 1: Table S1).

#### Major bleeding

**Fig. 5:**
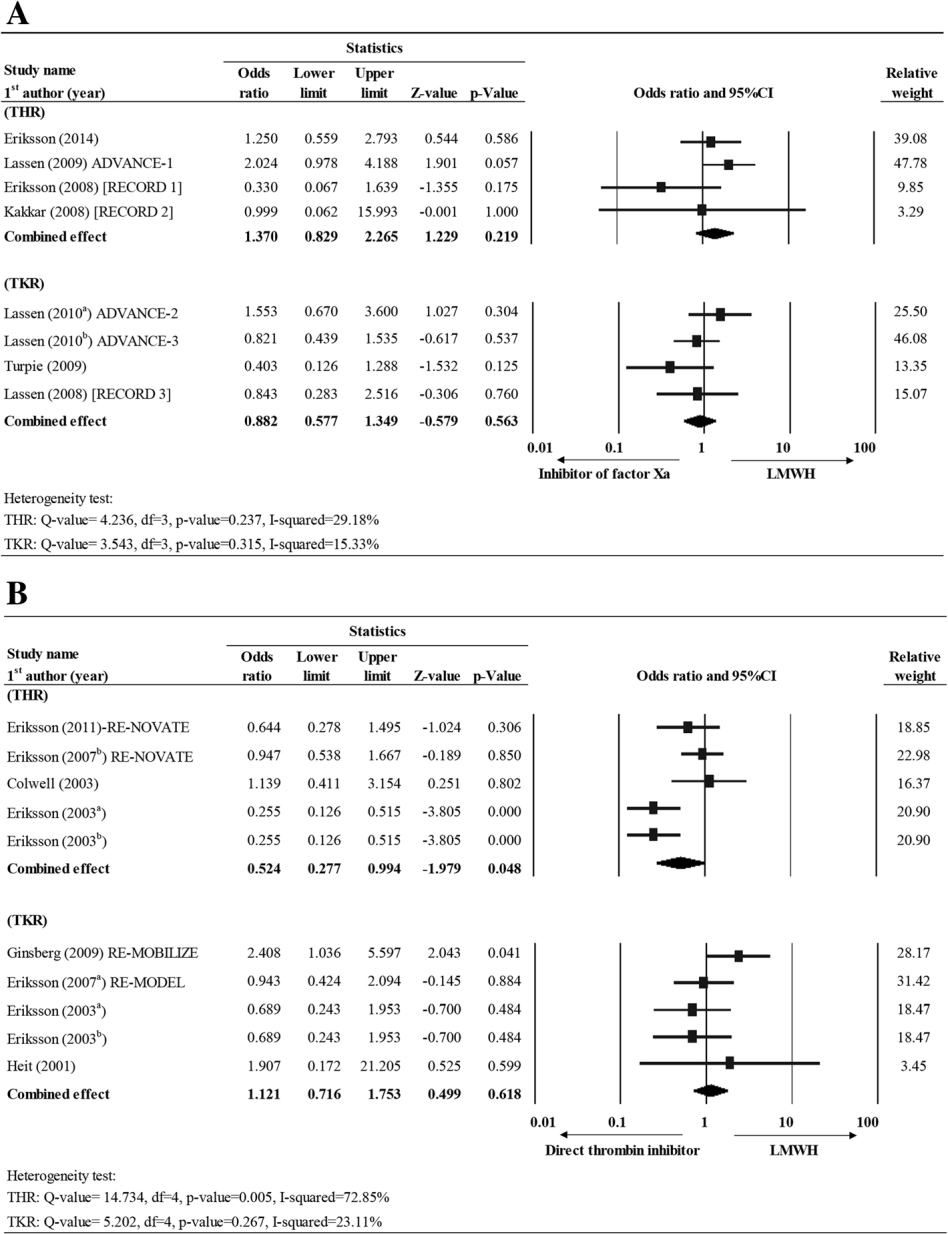
Forest plot for comparing the major bleeding rate between LMWH vs. (**a**) LMWH vs. inhibitor of factor Xa and (**b**) LMWH vs. direct thrombin inhibitor for THR and TKR patients. Abbreviations: CI, confidence interval; Lower limit, lower bound of the 95% CI; Upper limit, upper bound of the 95% CI

In Group II (LMWH vs. inhibitor of factor Xa), four studies [21, 28, 30, 31] for THR and four studies [24, 25, 27, 32] for TKR reported the incidence of major bleeding. Fixed effect model was used as low heterogeneity was observed for THR (Q value = 4.236, df = 3, *P* = 0.237, I-squared = 29.18%) and TKR (Q value = 3.543, df = 3, *P* = 0.315, I-squared = 15.33%). The overall effect size indicated that the chance of major bleeding was similar between types of treatment both for THR and TKR (THR: OR = 1.370, 95% CI = 0.829–2.265, *P* = 0.219; TKR: OR = 0.882, 95% CI = 0.577–1.349, *P* = 0.563) (Fig. 5a; Additional file 1: Table S1).

In Group III (LMWH vs. direct thrombin inhibitor), five studies reported major bleeding data for THR [23, 34–37] and five for TKR [10, 26, 33, 35, 37]. According to the heterogeneity test, random effects and fixed-effect models were applied for both THR and TKR patients, respectively (THR: Q-value = 14.73, df = 4, *P* = 0.005, I-squared = 72.85%; TKR: Q-value = 5.202, df = 4, *P* = 0.267, I-squared = 23.11%). The overall effect size showed that LMWH group was associated with a marginal lower rate of major bleeding than direct thrombin inhibitor for THR subjects (OR = 0.524, 95% CI = 0.277–0.994, *P* = 0.048) but similar likelihood of major bleeding for TKR subjects (OR = 1.121, 95% CI = 0.716–1.753, *p* = 0.618) (Fig. 5b; Additional file 1: Table S1).

##### Safety analyses

- Major bleeding

Overall incidence of major bleeding events was 1.27% [1.06, 1.48] in this population. In subgroup analysis, Enoxaparin treatment was associated with 1.32% [1.02, 1.63], Dabigatran 1.25% [0.68, 1.81], Rivaroxaban 2.02 [1.00, 3.04], Apixaban [0.70 [0.56, 0.84], and Ximelagatran with 0.93 [− 0.06, 1.91] (Additional file 1: Figure S6).

b) Reoperation rate

Overall reoperation rate was 0.26% [0.21, 0.31] in these patients. Treatment with Enoxaparin treatment was associated with 0.24% [0.17, 0.31], with Dabigatran 0.12% [0.05, 0.19], and with Rivaroxaban 0.28% [0.06, 0.49] reoperation rate (Additional file 1: Figure S7).

c) Mortality

Overall mortality during treatment was 0.13% [0.07, 0.19]. Mortality rate with Enoxaparin was 0.14% [0.04, 0.23], with Dabigatran 0.15% [− 0.01, 0.30], and with Rivaroxaban it was 0.19% [0.08, 0.31] (Additional file 1: Figure S8).

d) Other Adverse events

In this population, 4.31% [2.77, 5.86] patients discontinued treatment due to adverse side effects (4.57% [3.14, 6.00] with Enoxaparin and 5.53% [3.41, 7.66] with Dabigatran) (Additional file 1: Figure S9). Adverse reactions during treatment observed by one or more studies included fever, nausea, vomiting, diarrhea, constipation, urinary tract infections, wound infections, wound complications, wound secretion, wound hematoma, joint dislocation, hypotension, insomnia, edema, anemia, dizziness, headache, urinary problems, hemorrhage, blisters, pyrexia, cardiovascular events, myocardial infarction, and stroke.

Overall incidence of cardiovascular events was 0.36% [0.28, 0.44] (Enoxaparin 0.31% [0.20, 0.43], Dabigatran 1.05% [0.95, 1.15], Rivaroxaban 0.24 [− 0.04, 0.51], and Apixaban 0.15 [0.02, 0.28]) (Additional file 1: Figure S10). Overall incidence of stroke in these patients was 0.08% [0.06, 0.11] (Enoxaparin 0.06% [0.03, 0.10], Rivaroxaban 0.17% [0.09, 0.24], and Apixaban 0.04% [0.01, 0.07]) (Additional file 1: Figure S11).

### Sensitivity analysis

Sensitivity analyses were performed using a leave-one-out approach in which a meta-analysis for total VET (Additional file 1: Figure S1), total DVT (Additional file 1: Figure S2), PE (Additional file 1: Figure S3) and major bleeding (Additional file 1: Figure S4) were performed in which each study for a given analysis was left out in turn. The direction and magnitude of the combined estimates did not markedly differ with the removal of a single study, indicating that the meta-analysis had good reliability and that the data was not overly influenced by any study.

### Quality assessment

The results of quality assessment are shown in Additional file 1: Figure S5. In this figure, Panel A shows the potential risk of bias in an individual study, and Panel B shows the summary of bias for included studies. The most potential risk of bias came from attrition bias and selective reporting bias. Also, several studies failed to clearly indicate if they used an intent-to-treat in analysis. Overall, the included studies are of good quality.

## Discussion

Anticoagulants are routinely used to prevent deep vein thrombosis following TKR and THR to prevent DVT. However, the relative effectiveness of LMWH and other anticoagulants therapies in patients at risk for DVT has not been comprehensively studied. In the present study, the comparison of LMWH with placebo found that LMWH was associated with lower odds of VTE and DVT compared to placebo in THR subjects, suggesting that prophylactic treatment of patients with LMWH could significantly reduce the rate of VTE and DVT but the incidence of PE was similar between the two groups. Compared to factor Xa inhibitors (e.g. rivaroxaban, apixaban, darexaban), LMWH was associated with higher incidence of VTE in TKR subjects, but the odds of VTE was similar between treatment groups in THR subjects. LMWH was associated with higher likelihood of DVT in patients with either THR or TKR, suggesting that factor Xa inhibitors might be superior to LMWH in reducing the rate of VTE and DVT. However, both prophylactic treatments showed a similar chance of pulmonary embolism and major bleeding in patients with THR and TKR. The odds of VTE, DVT, PE were similar between LMWH and direct thrombin inhibitors (e.g. ximelagatran, dabigatran etexilate); although a marginal benefit in preventing major bleeding was observed for LMWH compared with direct thrombin therapies in patients with THR (*P* = 0.048). These results indicate that LMWH is an effective prophylactic agent for reducing VTE when it was compared with patients without prophylactic treatment. However, LMWH might be less effective than factor Xa inhibitors in reducing the risk of thromboembolic events. In general, LMWH showed effectiveness similar to direct thrombin inhibitors in reducing the risk of thromboembolic events as well as major bleeding.

The RCTs for comparing LMWH with placebo in THR or TKR subjects are rare in recent years. A prior systematic review by Hull et al. [40] assessed LMWH in comparison with placebo for the prevention of thrombosis in an out-patient setting in selective hip surgery subjects [40]. They found that compared to placebo, LMWH was associated with decreased episodes of DVT, proximal venous thrombosis, and symptomatic venous thrombosis. These findings support the extended out-of-hospital use of LMWH following hip surgery. A prior meta-analysis by Tasker et al. [41] assessed the in-patient clinical outcomes of LMWH compared to placebo in patients who had THR [41]. They found no difference between LMWH and placebo in affecting the risk of pulmonary embolism, other deaths, all-cause mortality, or major bleeding. They found that compared with placebo, LMWH reduced non-fatal PE at the expense of hematoma formation. Although, our study also assessed in-patient outcomes, it is difficult to compare our findings directly with those of Tasker et al. as we did not evaluate the relative effectiveness of LMWH and placebo with PE or major bleeding due to the limited number of studies reporting these outcomes.

Several systematic reviews and meta-analyses have evaluated the use of different anticoagulant therapies in TKR and THR subjects (see Additional file 1: Table S2) [8, 14, 42–56]. Consistent with the current study, the prior meta-analyses found that the factor Xa inhibitors, rivaroxaban and apixaban, have better anticoagulant effect as compared with the LMWH enoxaparin [42–44]. In contrast to our findings, the prior studies found enoxaparin had a higher incidence of major bleeding compared with some, but not all, of the factor Xa inhibitors. For example, the study of Gomez-Outes et al. [44] found that compared to enoxaparin, the relative risk of clinically relevant bleeding was higher with rivaroxaban, similar with dabigatran, and lower with apixaban. Gomez-Outes et al. concluded that the higher efficacy observed with the factor Xa inhibitors was generally associated with higher bleeding tendency than with LMWH [44]. The meta-analysis of Feng et al. [43] also found that rivaroxaban was associated with a higher bleeding rate [43]. In this meta-analysis, only those RCTs were included which compared the efficacy and safety of any oral direct factor Xa inhibitor with that of enoxaparin for elective THA or TKA. The oral direct factor Xa inhibitor included rivaroxaban, apixaban, darexaban, betrixaban, edoxaban and several developing drugs (e.g. BAY 59–7939, YM150, LY517717). In addition, several trials were open-label and therefore allocation concealment bias may have existed. The author also found that rivaroxaban had a higher bleeding rate, while apixaban and edoxaban did not show significantly higher bleeding risks [43].

Three previous meta-analyses compared the effectiveness of different direct thrombin inhibitors with enoxaparin [14, 45, 46]. In general, our results are similar to those of a few earlier studies which found that dabigatran was similar to enoxaparin with respect to VTE incidence. The same studies also found that the risk of major bleeding was similar between treatments. The meta-analysis of Cohen et al. found that ximelagatran had a significantly lower rate of VTE than with enoxaparin with no difference in bleeding rates [46]. Although, our meta-analysis did not assess individual direct thrombin inhibitors and so the findings are difficult to compare with the prior analyses, we did observe a potentially lower rate of major bleeding associated with LWMH.

The present study has several limitations that should be considered. In addition, the dosing regimens for the different therapies differed across studies. For example, three different regimens of enoxaparin (40 mg once daily or 20 mg or 30 mg bid) were used. A previous meta-analysis compared two different regimens of enoxaparin to oral anticoagulants (apixaban, dabigatran, and rivaroxaban) as thromboprophylaxis in elective TKR or THR [57]. An adjusted indirect comparison showed that bid 40 mg enoxaparin was significantly less effective than 30 mg bid in preventing VTE (relative risk 0.71, *P* < 0.001). The authors concluded that the use of once-daily 40 mg enoxaparin regimen as a control in clinical trials would lead to more favorable estimates of relative efficacy for the new oral anticoagulants than if enoxaparin 30 mg bid had been chosen as a comparator. Our study was able to assess the use of the different drugs in an in-patient setting only. It would be of interest to perform a similar analysis evaluating the long-term use to these therapies in an out-patient setting.

## Conclusions

This meta-analysis is the first to our knowledge to evaluate the overall relative effectiveness of LMWH by comparing with placebo control and two major classes of anticoagulants therapy (i.e., factor Xa inhibitors and direct thrombin inhibitor) to treat patient who had TKR or THR surgeries. The findings indicate that prophylactic treatment of patients with LMWH could significantly reduce the rate of VTE and DVT. However, the factor Xa inhibitors might have better anticoagulant effect as compared with the LMWH enoxaparin. Compared to direct thrombin inhibitors, LMWH have similar incidence of VTE, DVT and PE but lower incidence of major bleeding in THR or TKR subjects. In general, LMWH has similar effectiveness to factor Xa inhibitor and direct thrombin inhibitors with respect to clinical outcomes associated with anticoagulation therapy. Factor Xa inhibitors, such as rivaroxaban, is superior to enoxaparin in reducing symptomatic VTE but the trade-offs between thromboprophylaxis versus increased major bleeding should be considered.

### Key messages

- In comparison with patients without prophylaxis, low molecular weight heparin (LMWH) effectively reduces venous thromboembolism (VTE) and deep vein thrombosis (DVT) after total hip replacement (TKR).
- Compared to factor Xa inhibitors, LMWH may have higher incidence of VTE and DVT but similar rates of pulmonary embolism and major bleeding in THR or TKR subjects.
- In comparison with direct thrombin inhibitors, LMWH have similar incidence of VTE, DVT and pulmonary embolism but lower incidence of major bleeding in THR or TKR subjects.

## Additional file

Additional file 1: **Figure S1.** Sensitivity analysis using the leave-one-out approach of the influence of each study on the pooled estimate for comparing total VTE rate between LMWH vs. control (A) placebo, (B) inhibitor of factor Xa, and (C) direct thrombin inhibitor for THR and TKR patients. Abbreviations: CI, confidence interval; Lower limit, lower bound of the 95% CI; Upper limit, upper bound of the 95% CI. **Figure S2.** Sensitivity analysis using the leave-one-out approach of the influence of each study on the pooled estimate for comparing total DVT rate between LMWH vs. control (A) placebo, (B) inhibitor of factor Xa, and (C) direct thrombin inhibitor for THR and TKR patients. Abbreviations: CI, confidence interval; Lower limit, lower bound of the 95% CI; Upper limit, upper bound of the 95% CI. **Figure S3.** Sensitivity analysis using the leave-one-out approach of the influence of each study on the pooled estimate for comparing PE rate between LMWH vs. control (A) inhibitor of factor Xa, and (B) direct thrombin inhibitor for THR and TKR patients. Abbreviations: CI, confidence interval; Lower limit, lower bound of the 95% CI; Upper limit, upper bound of the 95% CI. **Figure S4.** Sensitivity analysis using the leave-one-out approach of the influence of each study on the pooled estimate for comparing major bleeding rate (A) inhibitor of factor Xa, and (B) direct thrombin inhibitor for THR and TKR patients. Abbreviations: CI, confidence interval; Lower limit, lower bound of the 95% CI; Upper limit, upper bound of the 95% CI. **Figure S5.** The results of quality assessment for (A) individual studies, and (B) the summary of bias for all included studies. **Figure S6.** Forest graph showing the incidence of major bleeding events. **Figure S7.** Forest graph showing reoperation rate in this population. **Figure S8.** Forest graph showing the immortality rate in this population. **Figure S9.** Forest graph showing the percentage of patients who discontinued treatment due to adverse reaction. **Figure S10.** Forest graph showing the incidence of cardiovascular events. **Figure S11.** Forest graph showing the incidence of stroke. **Table S1.** Summary of meta-analysis results. **Table S2.** Published systematic review and meta-analysis relating to thromboprophylasis after THR and TKR (DOCX 734 kb)

## Abbreviations

AAOS: American Association of Orthopedic Surgeons
ACCP: American College of Chest Physicians
DVT: deep vein thrombosis
MWH: low-molecular-weight heparin
RCTs: randomized controlled studies
THR: total hip replacement
TKR: received total knee
VTE: venous thromboembolism
