# Supplementary material for: Low molecular weight heparin versus other anti-thrombotic agents for prevention of venous thromboembolic events after total hip or total knee replacement surgery: a systematic review and meta-analysis
Source: BMC Musculoskelet Disord. 2018 Sep 8;19:322. doi: 10.1186/s12891-018-2215-3 (PMC6129001; doi:10.1186/s12891-018-2215-3)
Supplement: Supplementary file 1 — Figure S1. Sensitivity analysis using the leave-one-out approach of the influence of each study on the pooled estimate for comparing total VTE rate between LMWH vs. control (A) placebo, (B) inhibitor of factor Xa, and (C) direct thrombin inhibitor for THR and TKR patients. Abbreviations: CI, confidence interval; Lower limit, lower bound of the 95% CI; Upper limit, upper bound of the 95% CI. Figure S2. Sensitivity analysis using the leave-one-out approach of the influence of each study on the pooled estimate for comparing total DVT rate between LMWH vs. control (A) placebo, (B) inhibitor of factor Xa, and (C) direct thrombin inhibitor for THR and TKR patients. Abbreviations: CI, confidence interval; Lower limit, lower bound of the 95% CI; Upper limit, upper bound of the 95% CI. Figure S3. Sensitivity analysis using the leave-one-out approach of the influence of each study on the pooled estimate for comparing PE rate between LMWH vs. control (A) inhibitor of factor Xa, and (B) direct thrombin inhibitor for THR and TKR patients. Abbreviations: CI, confidence interval; Lower limit, lower bound of the 95% CI; Upper limit, upper bound of the 95% CI. Figure S4. Sensitivity analysis using the leave-one-out approach of the influence of each study on the pooled estimate for comparing major bleeding rate (A) inhibitor of factor Xa, and (B) direct thrombin inhibitor for THR and TKR patients. Abbreviations: CI, confidence interval; Lower limit, lower bound of the 95% CI; Upper limit, upper bound of the 95% CI. Figure S5. The results of quality assessment for (A) individual studies, and (B) the summary of bias for all included studies. Figure S6. Forest graph showing the incidence of major bleeding events. Figure S7. Forest graph showing reoperation rate in this population. Figure S8. Forest graph showing the immortality rate in this population. Figure S9. Forest graph showing the percentage of patients who discontinued treatment due to adverse reaction. Figure S10. Forest graph [file 12891_2018_2215_MOESM1_ESM.docx]

**Title Page**

**Low molecular weight heparin versus other anti-thrombotic agents for prevention of venous thromboembolic events after total hip or total knee replacement surgery: a systematic review and meta-analysis**

Xin Lu^1^, MD, Jin Lin^1,*^, MD

^1^Department of Orthopaedics, Peking Union Medical College Hospital, Chinese Academy of Medical Sciences and Peking Union Medical College, Beijing, China

*Corresponding author:

Dr. Jin Lin

Department of Orthopaedics, Peking Union Medical College Hospital, Chinese Academy of Medical Sciences and Peking Union Medical College, Beijing, China

Email: [jinlinjinlin0221@163.com](mailto:jinlinjinlin0221@163.com)

**Running title:** Heparin use in hip and knee replacement

**Supplementary Figures**

**Figure S1** Sensitivity analysis using the leave-one-out approach of the influence of each study on the pooled estimate for comparing total VTE rate between LMWH vs. control (A) placebo, (B) inhibitor of factor Xa, and (C) direct thrombin inhibitor for THR and TKR patients. Abbreviations: CI, confidence interval; Lower limit, lower bound of the 95% CI; Upper limit, upper bound of the 95% CI.

**Figure S2** Sensitivity analysis using the leave-one-out approach of the influence of each study on the pooled estimate for comparing total DVT rate between LMWH vs. control (A) placebo, (B) inhibitor of factor Xa, and (C) direct thrombin inhibitor for THR and TKR patients. Abbreviations: CI, confidence interval; Lower limit, lower bound of the 95% CI; Upper limit, upper bound of the 95% CI.

**Figure S3** Sensitivity analysis using the leave-one-out approach of the influence of each study on the pooled estimate for comparing PE rate between LMWH vs. control (A) inhibitor of factor Xa, and (B) direct thrombin inhibitor for THR and TKR patients. Abbreviations: CI, confidence interval; Lower limit, lower bound of the 95% CI; Upper limit, upper bound of the 95% CI.

**Figure S4** Sensitivity analysis using the leave-one-out approach of the influence of each study on the pooled estimate for comparing major bleeding rate (A) inhibitor of factor Xa, and (B) direct thrombin inhibitor for THR and TKR patients. Abbreviations: CI, confidence interval; Lower limit, lower bound of the 95% CI; Upper limit, upper bound of the 95% CI.

**Figure S5.** The results of quality assessment for (A) individual studies, and (B) the summary of bias for all included studies.

**Figure S6** Forest graph showing the incidence of major bleeding events.

**Figure S7** Forest graph showing reoperation rate in this population.

**Figure S8** Forest graph showing the immortality rate in this population.

**Figure S9** Forest graph showing the percentage of patients who discontinued treatment due to adverse reaction.

**Figure S10** Forest graph showing the incidence of cardiovascular events.

**Figure S11** Forest graph showing the incidence of stroke.

**Figure S1** Sensitivity analysis using the leave-one-out approach of the influence of each study on the pooled estimate for comparing total VTE rate between LMWH vs. control (A) placebo, (B) inhibitor of factor Xa, and (C) direct thrombin inhibitor for THR and TKR patients. Abbreviations: CI, confidence interval; Lower limit, lower bound of the 95% CI; Upper limit, upper bound of the 95% CI.

**Figure S2** Sensitivity analysis using the leave-one-out approach of the influence of each study on the pooled estimate for comparing total DVT rate between LMWH vs. control (A) placebo, (B) inhibitor of factor Xa, and (C) direct thrombin inhibitor for THR and TKR patients. Abbreviations: CI, confidence interval; Lower limit, lower bound of the 95% CI; Upper limit, upper bound of the 95% CI.

**Figure S3** Sensitivity analysis using the leave-one-out approach of the influence of each study on the pooled estimate for comparing PE rate between LMWH vs. control (A) inhibitor of factor Xa, and (B) direct thrombin inhibitor for THR and TKR patients. Abbreviations: CI, confidence interval; Lower limit, lower bound of the 95% CI; Upper limit, upper bound of the 95% CI.

**Figure S4** Sensitivity analysis using the leave-one-out approach of the influence of each study on the pooled estimate for comparing major bleeding rate (A) inhibitor of factor Xa, and (B) direct thrombin inhibitor for THR and TKR patients. Abbreviations: CI, confidence interval; Lower limit, lower bound of the 95% CI; Upper limit, upper bound of the 95% CI.

**Figure S5.** The results of quality assessment for (A) individual studies, and (B) the summary of bias for all included studies.


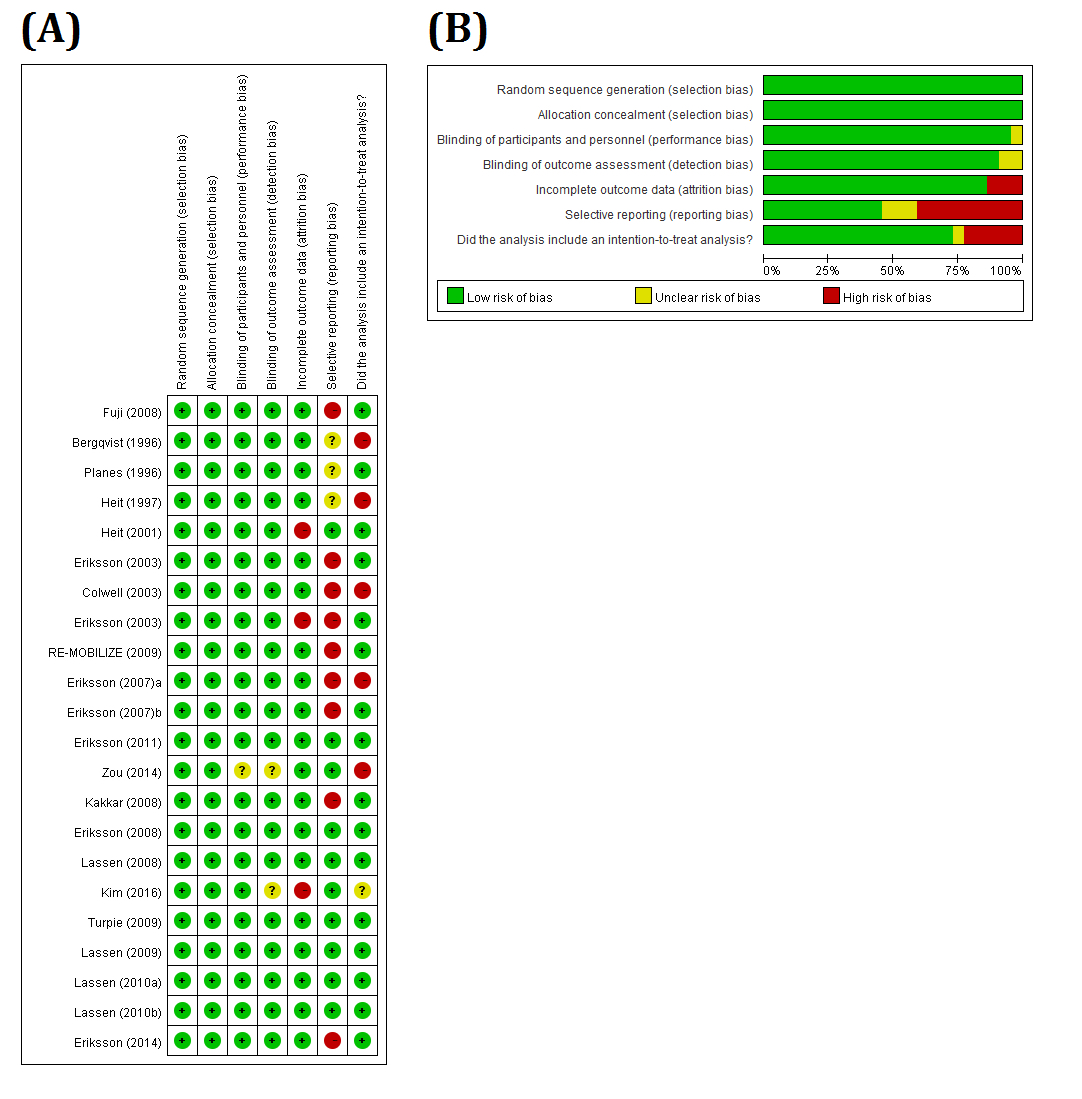


**Figure S6:** Forest graph showing the incidence of major bleeding events

**Figure S7:** Forest graph showing reoperation rate in this population

**Figure S8:** Forest graph showing the immortality rate in this population

**Figure S9:** Forest graph showing the percentage of patients who discontinued treatment due to adverse reaction.

**Figure S10:** Forest graph showing the incidence of cardiovascular events

**Figure S11:** Forest graph showing the incidence of stroke

**Supplemental Table 1.** Summary of meta-analysis results**.**

| **Outcomes** | **Subgroup** | **number of studies** | **Heterogeneity test** | **Odds ratio (95%CI)** | **p-value** |
| --- | --- | --- | --- | --- | --- |
| Total VTE | (I) LMWH vs. placebo (saline) |  |  |  |  |
|  | THR | 3 | Q-value= 2.922, df=2, p-value=0.232, I-squared=31.56% | 0.481 (0.338 - 0.685) | <.001 |
|  | TKR | 1 | No analysis | N/A | N/A |
|  | (II) LMWH vs. inhibitor of factor Xa |  |  |  |  |
|  | THR | 5 | Q-value= 37.097, df=4, p-value<.001, I-squared=89.22% | 2.023 (0.880 - 4.648) | 0.097 |
|  | TKR | 4 | Q-value= 0.906, df=3, p-value=0.824, I-squared=0% | 2.162 (1.513 - 3.089) | <.001 |
|  | (III) LMWH vs. direct thrombin inhibitor |  |  |  |  |
|  | THR | 5 | Q-value= 24.722, df=4, p-value<.001, I-squared=83.82% | 1.310 (0.757 - 2.267) | 0.335 |
|  | TKR | 4 | Q-value= 24.292, df=3, p-value<.001, I-squared=87.65% | 1.378 (0.817 - 2.323) | 0.229 |
| Total DVT | (I) LMWH vs. placebo (saline) |  |  |  |  |
|  | THR | 4 | Q-value= 4.060, df=3, p-value=0.255, I-squared=26.11% | 0.464 (0.332 - 0.647) | <.001 |
|  | TKR | 1 | No analysis | N/A | N/A |
|  | (II) LMWH vs. inhibitor of factor Xa |  |  |  |  |
|  | THR | 5 | Q-value= 35.701, df=4, p-value<.001, I-squared=88.80% | 2.351 (1.040 - 5.314) | 0.04 |
|  | TKR | 5 | Q-value= 13.523, df=4, p-value=0.009, I-squared=70.42% | 1.827 (1.352 - 2.468) | <.001 |
|  | (III) LMWH vs. direct thrombin inhibitor |  |  |  |  |
|  | THR | 3 | Q-value= 13.895, df=2, p-value=0.001, I-squared=85.61% | 1.004 (0.588 - 1.715) | 0.989 |
|  | TKR | 3 | Q-value= 16.857, df=2, p-value<.001, I-squared=88.14% | 1.155 (0.680 - 1.964) | 0.593 |
| PE | (I) LMWH vs. placebo (saline) |  |  |  |  |
|  | THR | 1 | No analysis | N/A | N/A |
|  | TKR | 1 | No analysis | N/A | N/A |
|  | (II) LMWH vs. inhibitor of factor Xa |  |  |  |  |
|  | THR | 5 | Q-value= 4.155, df=4, p-value=0.385, I-squared=3.74% | 0.554 (0.272 - 1.127) | 0.103 |
|  | TKR | 4 | Q-value= 4.600, df=3, p-value=0.204, I-squared=34.78% | 1.680 (0.724 - 3.896) | 0.227 |
|  | (III) LMWH vs. direct thrombin inhibitor |  |  |  |  |
|  | THR | 3 | Q-value= 0.440, df=2, p-value=0.802, I-squared=0% | 1.399 (0.524 - 3.732) | 0.503 |
|  | TKR | 4 | Q-value= 4.600, df=3, p-value=0.204, I-squared=34.78% | 1.588 (0.618 - 4.080) | 0.337 |
| Major bleeding | (I) LMWH vs. placebo (saline) |  |  |  |  |
|  | THR | 1 | No analysis | N/A | N/A |
|  | TKR | 1 | No analysis | N/A | N/A |
|  | (II) LMWH vs. inhibitor of factor Xa |  |  |  |  |
|  | THR | 4 | Q-value= 4.236, df=3, p-value=0.237, I-squared=29.18% | 1.370 (0.829 - 2.265) | 0.219 |
|  | TKR | 4 | Q-value= 3.543, df=3, p-value=0.315, I-squared=15.33% | 0.882 (0.577 - 1.349) | 0.563 |
|  | (III) LMWH vs. direct thrombin inhibitor |  |  |  |  |
|  | THR | 5 | Q-value= 14.734, df=4, p-value=0.005, I-squared=72.85% | 0.524 (0.277 - 0.994) | 0.048 |
|  | TKR | 5 | Q-value= 5.202, df=4, p-value=0.267, I-squared=23.11% | 1.121 (0.716 - 1.753) | 0.618 |

**Supplementary Table 2**. Published systematic review and meta-analysis relating to thromboprophylasis after THR and TKR

| **Author (Year)** | **Type of study** | **Number of trial included** | **Type of intervention** | **Anti-coagulants** | **Major conclusion** |
| --- | --- | --- | --- | --- | --- |
| Ning GZ (2016) [42] | Meta-analysis | 9 trials | Total hip or knee arthroplasty | Rivaroxaban vs. enoxaparin | Rivaroxaban was more beneficial than enoxaparin for preventing symptomatic DVT but increased the risk of major bleeding. |
| Feng W (2015) [43] | Systematic review & Meta-analysis | 20 trials | Total hip or knee arthroplasty | direct factor Xa inhibitors vs. enoxaparin | Rivaroxaban, apixaban and edoxaban showed a better anticoagulant effect, as compared with enoxaparin. Rivaroxaban had a higher bleeding rate, while apixaban and edoxaban did not show significantly higher bleeding risks. |
| Ma G (2015) [47] | Meta-analysis | 6 RCTs | Total knee replacement | Direct factor Xa inhibitors (rivaroxaban and apixaban) vs. enoxaparin | direct Xa inhibitors (rivaroxaban and apixaban) were more effective for prevention of VTE after total knee replacement as compared with enoxaparin, without increasing major bleeding risk. |
| Neumann I (2012) [48] | Systematic review & Meta-analysis | 22 trials | Total hip or knee replacement | Direct factor Xa inhibitors vs. LMWH | Compared with LMWH, lower doses of oral factor Xa inhibitors can achieve a small absolute risk reduction in symptomatic deep venous thrombosis without increasing bleeding. |
| Nieto JA (2012) [49] | Meta-analysis | 10 RCTs | Total hip or knee replacement | New oral anticoagulants (dabigatran, rivaroxaban, apixaban) vs. enoxaparin | New oral anticoagulants showed more efficacy and same safety when compared to the recommended dose of enoxaparin after THR and TKR without increasing the risk of major bleeding. |
| Gómez-Outes A (2012) [44] | Systematic review & Meta-analysis | 16 trials | Total hip or knee replacement | New oral anticoagulants (dabigatran, rivaroxaban, apixaban) vs. enoxaparin | Compared with enoxaparin, the risk of symptomatic venous thromboembolism was lower with new oral anticoagulants, but it associated with a higher bleeding tendency. |
| Li XM (2012) [50] | Meta-analysis | 4 RCTs | Total hip or knee replacement | Apixaban vs. enoxaparin | Apixaban was more effective than recommended dose of enoxaparin and had a similar incidence of bleeding events. |
| Raskob GE (2012) [51] | A pooled analysis of ADVANCE-2 and ADVANCE -3 trials | 2 RCTs | Total hip or knee replacement | Apixaban vs. enoxaparin | Apixaban 2.5 mg twice daily is more effective than enoxaparin 40 mg once daily without increased bleeding. |
| Huang J (2011) [52] | Meta-analysis | 3 RCTs | Total knee arthroplasty | Apixaban vs. enoxaparin | Apixaban was more effective than enoxaparin in decreasing the risk of proximal DVT, but no statistically significant differences were detected in PE. Apixaban was associated with a lower major bleeding rate than enoxaparin. |
| Kwong LM (2011) [8] | Systematic review of four RECORD trials | 4 RCTs | Total knee arthroplasty | Rivaroxaban vs. enoxaparin | Rivaroxaban is superiority over enoxaparin in reducing symptomatic VTE and all-cause mortality. There was no significant difference in major bleeding or in any other bleeding. |
| Cao YB (2010) [53] | Meta-analysis | 8 RCTs | Total hip or knee replacement | Rivaroxaban vs. enoxaparin | Rivaroxaban was more effective than the recommended dose of enoxaparin and showed a similar incidence of bleeding cases. |
| Huisman MV (2010) [14] | Meta-analysis | 6 RCTs | Total hip or knee replacement | New oral anticoagulants (dabigatran and rivaroxaban) vs. enoxaparin | Compared with dabigatran, enoxaparin had a similar risk of symptomatic venous thromboembolism and risk of bleeding. However, enoxaparin was less effective than rivaroxaban but had a lower risk of bleeding. |
| Melillo SN (2010) [54] | Systematic review of four RECORD trials |  | Total hip or knee replacement | Rivaroxaban vs. enoxaparin | Rivaroxaban use was significantly more effective for thromboprophylaxis compared to enoxaparin for DVT, PE, VTE; bleeding events occurred at statistically similar rates. |
| Eriksson B (2015) [55] | A pooled analysis of RE-NOVATE and RE-NOVATE II | 2 RCTs | Total hip replacement | Dabigatran vs. enoxaparin | Oral dabigatran 220 mg once daily was as effective as enoxaparin 40 mg once daily in reducing the risk of total VTE and all-cause mortality with a similar bleeding profile. |
| Friedman RJ (2010) [56] | A pooled analysis of RE- MOBILIZE, RE-NOVATE and RE-MODEL | 3 RCTs | Total hip or knee replacement | Dabigatran vs. enoxaparin | Oral dabigatran was as effective as subcutaneous enoxaparin in reducing the risk of major VTE and VTE-related mortality after hip or knee arthroplasty and has a similar bleeding profile. |
| Wolowacz SE (2009) [45] | A meta-analysis of RE- MOBILIZE, RE-NOVATE and RE-MODEL | 3 RCTs | Total hip or knee replacement | Dabigatran vs. enoxaparin | There were no significant differences between dabigatran etexilate and enoxaparin in total VET and bleed end points. |
| Cohen AT (2005) [46] | Meta-analysis | 6 RCTs | Total hip or knee replacement (major orthopaedic surgery) | Ximelagatran vs. enoxaparin | Compared with postoperative ximelagatran, LMWH had a significantly lower rate of VTE, with no significant difference in bleeding rate. |
